# PKN2 is involved in aggregation and spheroid formation of fibroblasts in suspension culture by regulating cell motility and *N-cadherin* expression

**DOI:** 10.1016/j.bbrep.2020.100895

**Published:** 2021-01-02

**Authors:** Koji Kubouchi, Hideyuki Mukai

**Affiliations:** a Graduate School of Medicine, Kobe University, Kobe, 650-0017, Japan; b Biosignal Research Center, Kobe University, Kobe, 657-8501, Japan

**Keywords:** Fibroblast, Aggregate, Compaction, Protein kinase, Cadherin, Transcription, KO, knockout, h, hour, Cre Adenovirus, Adenovirus for expression of Cre recombinase, CBB, coomassie brilliant blue, kDa, kilodalton

## Abstract

The role of Protein Kinase N2 (PKN2, also known as PRK2/PKNγ) in cell aggregate/spheroid formation in suspension culture was investigated using immortalized fibroblasts established from *PKN2*^flox/flox^ mouse embryos. *PKN2*^flox/flox^ cells formed cell aggregates in flat bottom low attachment well plates, such as 2% agar and poly-2-hydroxyethymethacrylate coated plates, however, Cre;*PKN2*^flox/flox^ cells in which PKN2 was depleted by the introduction of Cre-recombinase rarely formed aggregates. Time-lapse analysis revealed that the velocity of Cre;*PKN2*^flox/flox^ cell motility was significantly lower than that of *PKN2*^flox/flox^ in a low attachment flat-bottom plate, which likely resulted in a lower cell-cell contact frequency among Cre;*PKN2*^flox/flox^ cells. Conversely, Cre;*PKN2*^flox/flox^ cells could form initial cell aggregates in U-bottom low attachment well plates, however, the succeeding compaction process was delayed in Cre;*PKN2*^flox/flox^ cells with decreased roundness, although *PKN2*^flox/flox^ cells underwent compaction in a round shape spheroid within 24 h. Immunoblot analysis revealed that the preparation of the cell suspension from adherent conditions using trypsin/EDTA treatment significantly decreased the expression of N-cadherin in both *PKN2*^flox/flox^ and Cre;*PKN2*^flox/flox^ cells. The N-cadherin expression level recovered time-dependently; however, the recovery of N-cadherin was significantly delayed in Cre;*PKN2*^flox/flox^ cells compared to *PKN2*^flox/flox^ cells. Reverse transcription quantitative PCR revealed that *N-cadherin* mRNA in Cre;*PKN2*^flox/flox^ cells was significantly lower than that of *PKN2*^flox/flox^ cells. These results suggest that PKN2 controls the velocity of cell motility and the transcription of *N-cadherin* in fibroblasts, leading to cell aggregation and compaction for spheroid formation in suspension culture.

## Highlights

• PKN2 is involved in initial fibroblast aggregation by regulating cell motility.
• PKN2 is involved in compaction of fibroblasts in suspension.
• N-cadherin protein level seems to be a key element for compaction of fibroblasts.
• PKN2 controls transcription of *N-cadherin* mRNA in fibroblasts.

## 1 Introduction

Many types of cells can form aggregates and multicellular spheroids when cultured in suspension or in a non-adhesive environment. Cell aggregates/spheroids are regarded as a valuable tool in various studies *in vitro*. As examples, aggregates generated from tumours and stromal cells such as fibroblasts, endothelial cells and lymphocytes are employed in studies on cancer biology, inflammation, angiogenesis, and organoids [[1], [2], [3], [4]]. Although cell aggregates/spheroids are beneficial and promising from basic to clinical research, the mechanism of cell aggregate/spheroid formation has not yet been fully elucidated yet.

Protein kinase N (PKN) is a serine/threonine protein kinase with a catalytic domain homologous to protein kinase C and a unique regulatory region containing antiparallel coiled-coil (ACC) finger domains [5]. PKN is composed of three isoforms (PKN1, PKN2 and PKN3) derived from different genes in mammals [5]. These isoforms are ubiquitously expressed in mammalian tissues, albeit at differing amounts [6]. PKN1 kinase-negative mutant (PKN1[T778A]) mice develop to adulthood without apparent external abnormalities [7], however, the isolated primary fibroblasts from PKN1[T778A] mouse embryo showed impaired aggregates/spheroid formation in suspension culture with lower cell motility and surface expression of N-cadherin and integrins for unknown reasons [8]. PKN2 has been reported to regulate cell movement [9,10] and cell–cell adhesion [11,12]; therefore, we examined whether this PKN isoform is also involved in cell aggregate/spheroid formation. We tried to isolate primary fibroblasts from PKN2 KO embryos, and subject them to suspension culture. However, PKN2 KO mice are embryonically lethal by day 10.5 of gestation, and PKN2 KO primary fibroblasts isolated from embryos before death show severe growth retardation [13,14]; therefore we could not analyse aggregate/spheroid formation of these cells. Here, we isolated primary fibroblasts from *PKN2*^flox/flox^ mice and immortalized these cells. PKN2 was deleted by the expression of Cre recombinase in these cells, which were subjected to suspension culture. We report that PKN2 is important for fibroblast aggregate/spheroid formation in suspension culture through its involvement in cell motility and gene expression of *N-cadherin*. We also discuss the role of another isoform PKN3 in aggregate/spheroid formation.

## 2 Materials and methods

### 2.1 Preparation of immortalized *PKN2*^flox/flox^ cells

Primary mouse embryonic fibroblasts (MEFs) were derived from 14.5-day-old embryos of *PKN2*^flox/flox^ mice as described previously [13]. MEFs were incubated for 3 days with retrovirus expressing simian virus 40 large T antigen to immortalize cells in culture medium (Dulbecco's modified Eagle medium [DMEM; nacalai tesque] containing 10% fetal bovine serum [FBS; Gibco]) at 37 °C in 5% CO_2_ incubator. Immortalized *PKN2*^flox/flox^ cells were established in culture medium containing puromycin 0.8 μg/mL. Two weeks later, puromycin were removed. This study was approved by the Kobe university animal care and use committee.

### 2.2 Adenovirus infection

The adenovirus was prepared previously [13]. *PKN2*^flox/flox^ cells were treated with adenovirus in DMEM serum-free medium for 1 h at 37 °C in 5% CO_2_ incubator. One hour later, the medium containing adenovirus was removed and then cells were cultured in culture medium for the indicated time.

### 2.3 Preparation of cell suspension

Cells were treated with trypsin-EDTA (Trypsin 0.05% (w/v) and EDTA 0.02% (w/v) in PBS) for a few minutes in CO_2_ incubator. Then, cells were suspended with culture medium. Cell suspension was plated in 2% agar coated flat bottom well plate and U-bottom ultra-low attachment (PrimeSurface®) well plate. When cell suspension was prepared with thermal detachment cell cultureware (Cepallet®), cells plated in Cepallet® were incubated for 1 h at 4 °C and then suspended cells were collected.

### 2.4 Morphological analysis of cells

Cell morphology, velocity of cell motility, cell area and roundness were analysed as described previously [8]. The roundness indicates the circularity of the projected area of the cell aggregate.

### 2.5 Stability of mRNA

Cells were treated with actinomycin D (Wako) 2 μg/mL in culture medium at 37 °C in 5% CO_2_ incubator. Total RNA was purified from the cells at the indicated time after addition of actinomycin D.

### 2.6 Immunoblotting

Samples were subjected to 8%–12.5% SDS-PAGE and separated products were subsequently transferred to a polyvinylidene difluoride membrane. The membrane was blocked with Blocking One (nacalai tesque) for 1 h at room temperature. The membrane was then incubated in Signal Enhancer HIKARI (nacalai tesque) solution A and the primary antibody for overnight at 4 °C. The membrane was washed in TBS-T before incubating the blot in solution B containing the secondary antibody conjugated to horseradish peroxidase at 1:5000 dilution for 1 h. Then, the membrane was washed in TBS-T. Development and intensity analysis were performed as described previously [13]. Primary antibodies are shown in Supplementary Table 1.

### 2.7 RT-qPCR (reverse transcription quantitative polymerase chain reaction)

Total RNA of cells was isolated by using an RNeasy mini kit (QIAGEN). Preparation of cDNA and qPCR were performed as described previously [6]. Primers for RT-qPCR are shown in Supplementary Table 2. Delta-delta CT method was used to calculate relative fold expression of mRNA.

### 2.8 Statistical analysis

Data presented in the figures and text represent mean ± SEM of independent experiments. Each experiment was repeated at least three times. Statistical significance was calculated using paired *t*-test, unpaired *t*-test or repeated measures ANOVA with P value < 0.05 indicated in the figure legends.

## 3 Results

PKN2 depletion suppresses the velocity of cell motility and cell aggregation in suspension culture.

**Fig. 1:**
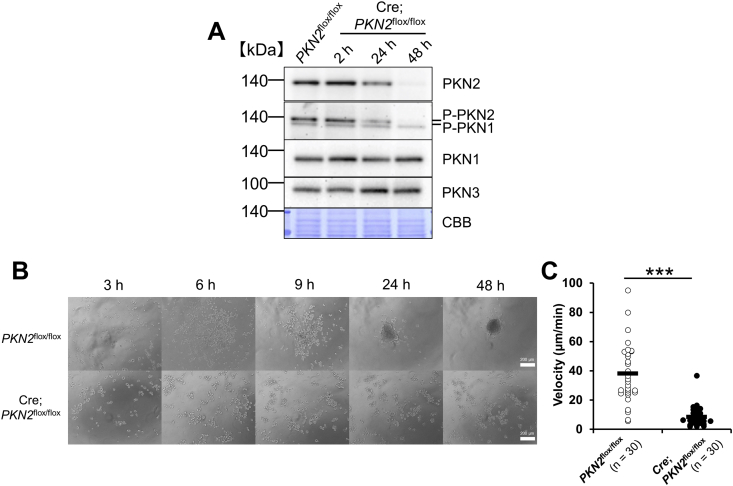
PKN2 depletion suppresses velocity of cell motility and cell aggregation. (A) Expression of PKN isoforms. *PKN2*^flox/flox^ cells were incubated for the indicated time after treatment with Cre adenovirus (“Cre; *PKN2*^flox/f^^lox^”), *PKN2*^flox/flox^ cells were incubated for 48 h after mock adenovirus treatment (“*PKN2*^flox/flox^”). After removing culture medium, whole cell lysate was subjected to immunoblotting. (B) Morphological change of cells. *PKN2*^flox/flox^ cells were incubated for 48 h after treatment with mock adenovirus (“*PKN2*^flox/flox^”) and Cre adenovirus (“Cre;*PKN2*^flox/flox^”), respectively. Cells were collected using trypsin-EDTA treatment and then plated in 2% agar coated flat bottom well plate. Phase contrast images were taken at the indicated time after plating cells. (C) Velocity of cell motility. *PKN2*^flox/flox^ cells were incubated for 48 h after treatment with mock adenovirus (“*PKN2*^flox/flox^”) and Cre adenovirus (“Cre;*PKN2*^flox/flox^”), respectively. Cells were collected using trypsin-EDTA treatment and then plated in poly hema-coated flat bottom well plate. Cell velocity was measured by tracking cells in time-lapse movie for the first 1 h. Data were analysed using unpaired *t*-test. ***P < 0.001. Movie_ *PKN2*^flox/flox^ and Movie_ Cre;*PKN2*^flox/flox^. Time-lapse movies showed the cell aggregation of *PKN2*^flox/flox^ (Movie 1.avi) and Cre;*PKN2*^flox/flox^ (Movie 2.avi) cells in suspension culture in poly hema-coated flat bottom well plate. Movies were taken from 30 min after plating and continued for 6 h.

To analyse the role of PKN2 in suspension culture, we established immortalized fibroblasts (*PKN2*^flox/flox^ cells) from *PKN2*^flox/flox^ MEFs [13]. PKN2 protein expression, morphology, and the cell growth rate of *PKN2*^flox/flox^ cells were almost similar to those of immortalized wild-type cells (data not shown). The ectopic expression of Cre recombinase in *PKN2*^flox/flox^ cells using adenovirus decreased the PKN2 protein level time-dependently, reaching undetectable levels within 48 h after adenovirus treatment (Cre;*PKN2*^flox/flox^). Neither PKN1 nor PKN3 protein expression was affected by PKN2 depletion (Fig. 1A). To observe cell morphology in suspension culture, *PKN2*^flox/flox^ and Cre;*PKN2*^flox/flox^ cells were suspended from adherent culture conditions using trypsin-EDTA treatment and then placed in a 2% agar coated flat bottom plate. *PKN2*^flox/flox^ cells formed aggregates within 6 h, time-dependently, and finally formed a round spheroid at 48 h after plating the cells (Fig. 1B). However, Cre;*PKN2*^flox/flox^ cells could not assemble and form a round spheroid at 48 h after plating the cells. Time-lapse imaging revealed that the velocity of Cre;*PKN2*^flox/flox^ cell motility was significantly lower than that of *PKN2*^flox/flox^ cells in a poly hema-coated flat bottom plate (Fig. 1C; Movie_*PKN2*^flox/flox^ and Movie_ Cre;*PKN2*^flox/flox^). These data suggest that PKN2 expression is necessary for the cell motility and aggregation of suspension cells.

PKN2 depletion impairs the compaction for a round spheroid formation in U-bottom ultra-low attachment plate.

**Fig. 2:**
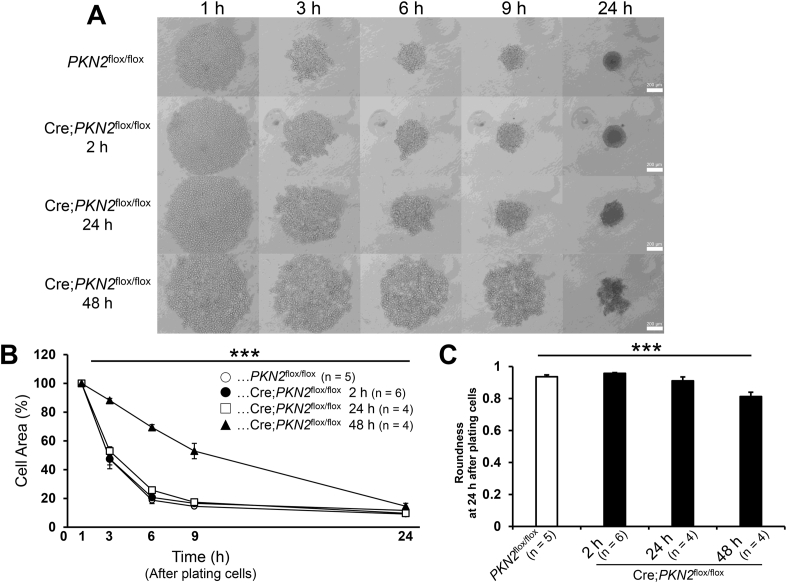
PKN2 depletion impairs compaction for a round spheroid formation. *PKN2*^flox/flox^ cells were incubated for the indicated time after treatment with Cre adenovirus (“Cre;*PKN2*^flox/flox^”). *PKN2*^flox/flox^ cells were incubated for 48 h after mock adenovirus treatment (“*PKN2*^flox/flox^”). Cells were collected using trypsin-EDTA treatment and then plated in U-bottom PrimeSurface® plate. (A) Morphological change of cell aggregates. Phase contrast images were taken at the indicated time after plating cells. (B) Rate of cell compaction. Area covered by cell aggregates at 1 h after plating cells is regarded as 100%. Data were analysed using repeated measures ANOVA. ***P < 0.001. (C) Roundness of the cell aggregate. Data were analysed using repeated measures ANOVA. ***P < 0.001.

The generation of a multicellular spheroid of fibroblasts in suspension culture is regarded as a stepwise process. Cells are assembled into loose cell aggregates and then undergo time-dependent formation of tight spheroids, i.e. the compaction stage [15]. An impaired initial assembly stage in Cre;*PKN2*^flox/flox^ cells was observed in suspension culture as shown in Fig. 1B. To investigate whether PKN2 plays a role in the compaction stage in suspension culture, *PKN2*^flox/flox^ and Cre;*PKN2*^flox/flox^ cells were plated in a U-bottom ultra-low attachment (PrimeSurface®) well plate. *PKN2*^flox/flox^ cells underwent compaction for a round spheroid, time-dependently (Fig. 2A). However, the compaction rate of Cre;*PKN2*^flox/flox^ cells was delayed along with length of incubation time after adenovirus treatment (Fig. 2B), suggesting that compaction is impaired depending on the degree of PKN2 depletion (Fig. 1A). The area size of the compacted Cre;*PKN2*^flox/flox^ cells finally reached that of the *PKN2*^flox/flox^ cell at 24 h after plating cells in the U-bottom PrimeSurface® plate (Fig. 2B). However, the shape of Cre;*PKN2*^flox/flox^ cell spheroids showed significantly less roundness than that of *PKN2*^flox/flox^ cell spheroids (Fig. 2C). These data suggest that PKN2 is involved in cell compaction for a round spheroid in suspension culture.

PKN2 deficient cells suspended without trypsin-EDTA treatment smoothly undergo compaction in a U-bottom ultra-low attachment plate.

**Fig. 3:**
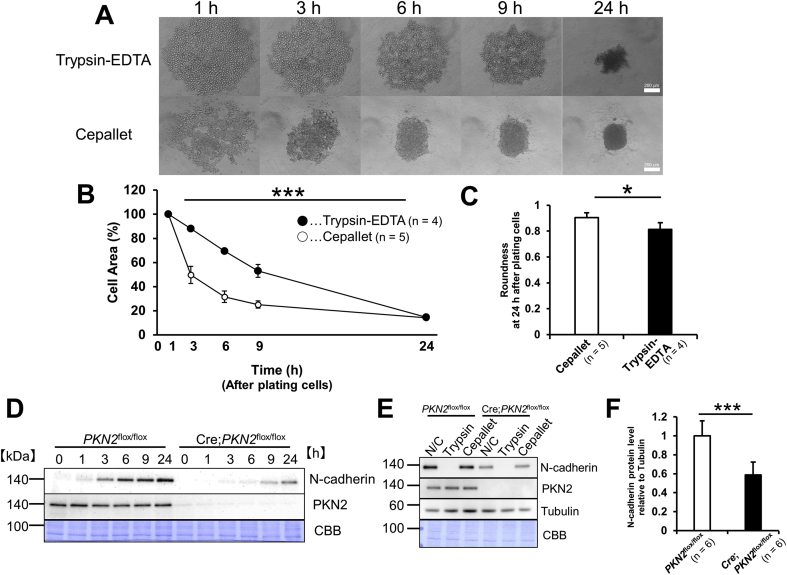
PKN2 deficient cells suspended using Cepallet smoothly undergo compaction. (A) Morphological change of cell aggregates. *PKN2*^flox/flox^ cells were incubated for 48 h after treatment with Cre adenovirus. Cells were collected using trypsin-EDTA treatment (“Trypsin-EDTA”) and using Cepallet® plate (“Cepallet”). Cells were plated in U-bottom PrimeSurface® plate. Phase contrast images were taken at the indicated time after plating cells. (B) Rate of cell compaction. Area covered by cell aggregates at 1 h after plating cells is regarded as 100%. Data were analysed using repeated measures ANOVA. ***P < 0.001. (C) Roundness of the cell aggregate. Data were analysed using repeated measures ANOVA. *P < 0.05. (D) Time course after trypsin-EDTA treatment. *PKN2*^flox/flox^ cells were incubated for 48 h after treatment with mock adenovirus (“*PKN2*^flox/flox^”) and Cre adenovirus (“Cre;*PKN2*^flox/flox^”), respectively. Cells collected using trypsin-EDTA treatment were incubated for the indicated time in 2% ager coated flat bottom well plate, and the whole cell lysate was subjected to immunoblotting. (E) Effect of different procedure to prepare cell suspension on N-cadherin. *PKN2*^flox/flox^ cells were incubated for 48 h after treatment with mock adenovirus (“*PKN2*^flox/flox^”) and Cre adenovirus (“Cre;*PKN2*^flox/flox^”), respectively. Whole cell lysates of adherent cells (Negative Control: “N/C”), cells collected using trypsin-EDTA treatment (“Trypsin”) and cells collected using Cepallet® plate (“Cepallet”) were subjected to immunoblotting. (F) N-cadherin protein content in adherent cells. The intensities of the N-cadherin and Tubulin bands as negative control of *PKN2*^flox/flox^ and Cre;*PKN2*^flox/flox^ cells were measured. Data were analysed by unpaired *t*-test. ***P < 0.001.

Cell surface proteins are critically involved in aggregate/spheroid formation in suspension culture [4,15,16]. It can be speculated that the expression of some cell surface proteins is different between Cre;*PKN2*^flox/flox^ and *PKN2*^flox/flox^ cells prepared for suspension culture. Cell suspensions were prepared from adherent cells using a trypsin-EDTA treatment. The trypsin-EDTA treatment is known to affect the expression of cell surface proteins [17,18]; thus, we then applied thermal detachment cell cultureware (Cepallet®) to prepare the cell suspensions to avoid the effect of trypsin-EDTA treatment on cell surface proteins as described in Materals and methods. The prepared cell suspensions were placed in U-bottom PrimeSurface® plate. Morphological analysis revealed that compaction of *PKN2* deficient cell suspension prepared using Cepallet proceeded (Fig. 3A), almost comparably to *PKN2*^flox/flox^ cells, which was much faster and yielded more roundness than that of *PKN2* deficient cell suspension prepared using trypsin-EDTA (Fig. 3B and C). Next we tried to identify the cell surface proteins responsible for the difference in the compaction process between *PKN2*^flox/flox^ and Cre;*PKN2*^flox/flox^ cells uncovered using trypsin-EDTA treatment. One of the cell surface proteins, N-cadherin is demonstrated to be destroyed when using trypsin-EDTA treatment [17] and is responsible for aggregate/spheroid formation, especially cell lines with low or no expression of E-cadherin [16,17,19]. Fibroblasts express N-cadherin, whereas E- and P-cadherins are undetectable as reported [20]. Thus, we compared the expression of N-cadherin in Cre;*PKN2*^flox/flox^ and *PKN2*^flox/flox^ cell suspension. Immunoblotting revealed that N-cadherin protein was not detected immediately after the preparation of the cell suspension using trypsin-EDTA treatment; however, it increased time-dependently in both types of cells (Fig. 3D). Notably, the amount of N-cadherin was much lower in Cre;*PKN2*^flox/flox^ cells than in *PKN2*^flox/flox^ cells at each time point, especially until 6 h (Fig. 3D). Conversely, N-cadherin in the cell suspension even immediately after preparation using Cepallet®, was almost equal to that of adherent cells before suspension for each Cre;*PKN2*^flox/flox^ and *PKN2*^flox/flox^ genotype (Fig. 3E). Immunochemical analysis showed that the N-cadherin protein abundance in Cre;*PKN2*^flox/flox^ cells was approximately more than half of that in *PKN2*^flox/flox^ cells in adherent condition (Fig. 3F). These results indicate the possibility that the amount of N-cadherin is key for the compaction process difference observed between *PKN2*^flox/flox^ and Cre;*PKN2*^flox/flox^ cells, and that PKN2 is involved in the expression of N-cadherin not only during the recovery phase after trypsin-EDTA treatment, but also in normal adherent condition.

PKN2 is necessary to maintain N-cadherin gene expression.

**Fig. 4:**
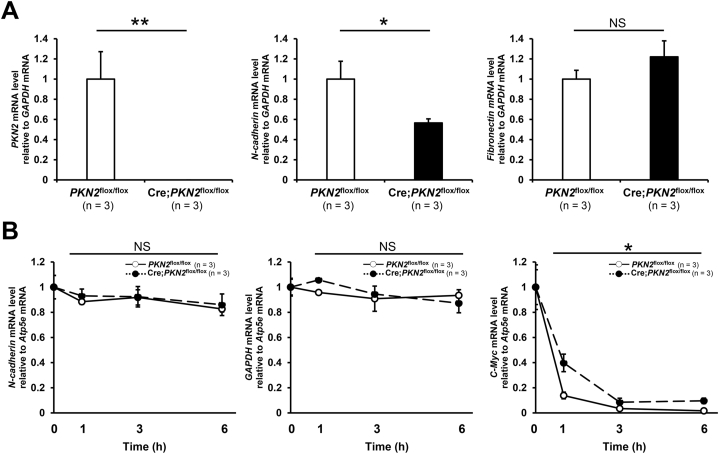
PKN2 is necessary to maintain *N-cadherin* mRNA expression. *PKN2*^flox/flox^ cells were incubated for 48 h after treatment with mock adenovirus (“*PKN2*^flox/flox^”) and Cre adenovirus (“Cre;*PKN2*^flox/flox^”), respectively. (A) mRNA expression. Cells were collected using trypsin-EDTA treatment and then incubated for 3 h in 2% agar coated flat bottom well plate. Expression of *GAPDH* mRNA was used as an internal control for normalization. Data were analysed by unpaired *t*-test. *P < 0.05, **P < 0.01, NS, not significant. (B) Stability of mRNA. mRNA of the *PKN2*^flox/flox^ cells (white circle) and Cre;*PKN2*^flox/flox^ cells (black circle) treated with actinomycin D was measured. Expression of *Atp5e* mRNA was used as an internal control for normalization. Data were analysed using repeated measures ANOVA. *P < 0.05, NS, not significant.

To clarify the mechanism by which PKN2 depletion decreases N-cadherin protein expression, we compared the *N-cadherin* mRNA expression between *PKN2*^flox/flox^ and Cre;*PKN2*^flox/flox^ cells. RT-qPCR analysis revealed that *PKN2* mRNA was undetectable in Cre;*PKN2*^flox/flox^ cell suspension, as expected (Fig. 4A). *N-cadherin* mRNA in Cre;*PKN2*^flox/flox^ cell suspension was approximately half of that in *PKN2*^flox/flox^ cell suspension, whereas the mRNA level of fibronectin, another mesenchymal marker, was not decreased by PKN2 depletion. *N-cadherin* mRNA of adherent Cre;*PKN2*^flox/flox^ cells was also decreased compared with adherent *PKN2*^flox/flox^ cells (Supplementary Fig. 1). These results suggest that PKN2 is involved in maintaining *N-cadherin* mRNA expression irrespective of the cell culture conditions. To examine the stability of *N-cadherin* mRNA, we inhibited transcription in both adherent *PKN2*^flox/flox^ and Cre;*PKN2*^flox/flox^ cells using actinomycin D and measured the RNA levels over a period of 6 h. We validated the transcriptional blocking methodology by determining the stability of the *c-Myc* oncogene mRNA, which is known to have a very short half-life [21]. *Atp5e* mRNA which is known to be stable [21] was used as an internal control. The stability of *N-cadherin* mRNA was not affected by PKN2 depletion although *c-Myc* mRNA of Cre;*PKN2*^flox/flox^ cells was more stable than that of *PKN2*^flox/flox^ cells (Fig. 4B). These results suggest that PKN2 is involved in the transcription, but not in the stability of *N-cadherin* mRNA.

## 4 Discussion

PKN2 depletion decreased the velocity of cell motility, which seems to suppress the initial cell aggregation due to a lower cell-cell contact frequency. How does PKN2 affect cell motility in suspension culture? In general, migration of mammalian cells without flagellum in suspension culture, completely in the absence of adhesion to a surface, has not been sufficiently studied so far. Aoun et al. have recently described the primary lymphocyte mechanism of “swimming”, which is mainly due to rearward and inhomogeneous treadmilling of the cell external membrane, leading to a ‘‘paddling’’ of transmembrane proteins linked to and advected by the cell cortex [22]. Whether other cell types such as fibroblasts can “swim” is still a question; however, if this mode of motility is relevant, actin network contractility or actin polymerisation seems to play major roles under Rho family GTPases [23]. PKN2 is reported to bind to Rho family GTPases and to be involved in actin cytoskeletal regulation [5]. Therefore, PKN2 likely contributes to the promotion of cell motility in suspension culture under the control of Rho family GTPases.

We hypothesized that N-cadherin abundance is a key factor accounting for the difference in the compaction process between *PKN2*^flox/flox^ and Cre;*PKN2*^flox/flox^ cells uncovered using trypsin-EDTA treatment, based on the following evidences: i) The compaction of Cre;*PKN2*^flox/flox^ cells was delayed and irregular, with a much lower amount of N-cadherin compared to the *PKN2*^flox/flox^ cells, when prepared using trypsin-EDTA. ii) The compaction of Cre;*PKN2*^flox/flox^ cells was almost complete, keeping more than half of the amount of N-cadherin compared to the *PKN2*^flox/flox^ cells, when prepared using Cepallet. iii) MEFs prepared from *PKN3* KO (*PKN3*^−/−^) mice could form aggregates/spheroids in suspension culture as well as wild type cells, having a comparable amount of N-cadherin to that of wild-type cells, even after trypsin-EDTA treatment (Supplementary Fig. 2). iv) The amounts of integrin α5 and β1 were intact in Cre;*PKN2*^flox/flox^ cells (Supplementary Fig. 3) although these integrins have been previously reported to be responsible for cell aggregate/spheroid formation of fibroblasts [15]. Homozygous N-cadherin null mouse embryos die by day 10 of gestation; however, heterozygous mice with an approximately ~50% abundance of N-cadherin relative to the wild-type, have a normal size and fecundity without displaying any obvious abnormalities [24,25]. Therefore, the existence of ~50% of N-cadherin seems to be sufficient for accomplishing a fundamental biological role, which also supports our above hypothesis.

*N-cadherin* mRNA expression was decreased in PKN2 depleted cells. How does PKN2 control the expression of *N-cadherin*? Previous studies demonstrated that the 5′-flanking region of the *N-cadherin* gene contains several putative transcriptional regulatory elements, such as E-box and Sp1 [26]. Slug, ZEB1 and Twist1 transcriptional factors are known to bind to E-box elements and promote *N-cadherin* expression [[27], [28], [29]]. However, mRNA levels of these transcriptional factors were not decreased in Cre;*PKN2*^flox/flox^ cells compared to *PKN2*^flox/flox^ cells (Supplementary Fig. 4). c-Myc also binds to E-box element and the overexpression of c-Myc decreases N-cadherin expression in hematopoietic stem cells [30], raising the possibility that an increased c-Myc level in Cre;*PKN2*^flox/flox^ cells (Supplementary Fig. 4) suppresses *N-cadherin* expression. So far there have been accumulated reports regarding the involvement of PKN2 in the regulation of the transcription of some genes. A few potential links between PKN2 and N-cadherin expression can be raised as follows: i) PKN2 is involved in the transcriptional activation of serum response factor (SRF) and its coactivators GATA, MEF2, and Myocardin-Related Transcription Factor A [[31], [32], [33], [34]]. SRF increases the N-cadherin expression [35]. ii) PKN2 promotes AP-1 mediated transcriptional upregulation under CD44-Rac1 signaling [36]. AP-1 positively regulates the expression of *N-cadherin* gene expression [37].

Here, we revealed the role of PKN2 in cell-cell interactions of fibroblasts in suspension, which were not evident in normal adherent culture conditions. A cell suspension of fibroblasts is speculated to occur *in vivo* during the destruction of the connective tissue and liberation of fibroblasts during inflammation, cancer, and wound healing. The role of PKN2 in physiological relevance in such situations awaits further studies.

## Declaration of competing interest

The authors declare that they have no known competing financial interests or personal relationships that could have appeared to influence the work reported in this paper.
