# Supplementary material for: PKN2 is involved in aggregation and spheroid formation of fibroblasts in suspension culture by regulating cell motility and N-cadherin expression
Source: Biochem Biophys Rep. 2021 Jan 2;25:100895. doi: 10.1016/j.bbrep.2020.100895 (PMC7787963; doi:10.1016/j.bbrep.2020.100895)
Supplement: Multimedia component 1 [file mmc1.zip › Kubouchi_supplementary information.docx]

**Supplementary information**

**Table 1. Primary antibodies for immunoblotting.**

| **Antibody** | **Target** | **Notation in Figure** |
| --- | --- | --- |
| αC6 | PKN1 | PKN1 |
| Anti-PKN2 mouse monoclonal antibody | PKN2 | PKN2 |
| αNUS | PKN3 | PKN3 |
| Anti-phospho PKN1 (Thr774)/PKN2 (Thr816) rabbit polyclonal antibodies | PKN1 and PKN2 phosphorylated at Thr774 or Thr816, respectively. | P-PKN1 and P-PKN2 |
| Anti-α-Tubulin mouse monoclonal antibody | α-Tubulin | Tubulin |
| Anti-N-cadherin mouse monoclonal antibody | N-cadherin | N-cadherin |
| Anti-integrin α5 rabbit polyclonal antibodies | integrin α5 | integrin α5 |
| Anti-integrin β1 rabbit polyclonal antibodies | integrin β1 | integrin β1 |

The polyclonal antibody αC6 against PKN1 was prepared as described [1]. The polyclonal antibody αNUS against PKN3 was prepared as described [2]. The anti-phospho PKN1 (Thr774)/PKN2 (Thr816) rabbit polyclonal antibody (#2611), anti-α-Tubulin mouse monoclonal antibody (#3873), anti-integrin α5 rabbit polyclonal antibody (CST #4705), anti-integrin β1 rabbit polyclonal antibody (CST #4706) were purchased from Cell Signaling Technology. The anti-PKN2 mouse monoclonal antibody (ab119936) was purchased from abcam. The anti-N-cadherin mouse monoclonal antibody (610920) was purchased from BD transduction.

**Table 2. Primers for RT-qPCR.**

| **Gene** | **Forward primer** | **Reverse primer** |
| --- | --- | --- |
| *PKN2* | GTGGGGCCTTGGTGTACTTA | TGGATACCTTACTTCGTCATTTACA |
| *N-cadherin* | GCCATCATCGCTATCCTTCT | CCGTTTCATCCATACCACAAA |
| *Fibronectin* | GGAATGGACCTGCAAACCTA | GTAGGGCTTTTCCCAGGTCT |
| *c-Myc* | CTGCTGTCCTCCGAGTCCT | GCCTCTTCTCCACAGACACC |
| *GAPDH* | TCTCTGCTCCTCCCTGTTCC | GTTCACACCGACCTTCACCA |
| *Atp5e* | CCGGTTTGAGGCTACTCTGA | AGATCTGGGAAAACCGGATG |
| *Slug* | CATTGCCTTGTGTCTGCAAG | AGAAAGGCTTTTCCCCAGTG |
| *ZEB1* | CTGGACAGATTCCAGGACAGT | TCGGTTTTTGTAGGTATGTTTACTGA |
| *Twist1* | AGCTACGCCTTCTCCGTCT | CTAGTGGGACGCGGACAT |

**Supplementary Fig. 1. PKN2 depletion decreases *N-cadherin* mRNA expression in adherent cells.**

*PKN2*^flox/flox^ cells were incubated for 48 h after treatment with mock adenovirus (“*PKN2*^flox/flox^”) and Cre adenovirus (“Cre;*PKN2*^flox/flox^”), respectively. mRNA expression of *PKN2*, *N-cadherin* and *Fibronectin* was measured by RT-qPCR. Relative gene expression was calculated by delta-delta CT method. Expression of *GAPDH* mRNA was used as an internal control for normalization. Data were analyzed by unpaired t-test. n = 3. *P < 0.05, **P < 0.01, NS, not significant.

**Supplementary Fig. 2. *PKN3* KO does not affect cell aggregate/spheroid formation and protein expression of N-cadherin and integrins in fibroblasts.**

Wild-type (“WT”) and *PKN3* KO (“*PKN3* KO”) primary MEFs were derived from 14.5-day-old embryos of wild-type and PKN3 KO mice as described previously [2]. The primary MEFs were immortalized with the same method as *PKN2*^flox/flox^ cells. (A, B) Morphological change of cells. Cells were collected using trypsin-EDTA treatment and then plated in a 2% agar coated flat bottom well plate (A) and a U-bottom PrimeSurface^®^ plate (B). Phase contrast images were taken at the indicated time after plating cells. (C) N-cadherin and integrin expression in adherent cells. After removing culture medium, whole cell lysate was subjected to immunoblotting. (D) Time course of N-cadherin protein expression in suspension culture. Cells were collected using trypsin-EDTA treatment and then incubated for the indicated time in a 2% ager coated flat bottom well plate. The whole cell lysate was subjected to immunoblotting.

**Supplementary Fig. 3. Protein expression of integrin α5 and β1 are not affected by PKN2 depletion.**

*PKN2*^flox/flox^ cells were incubated for 48 h after treatment with mock adenovirus (“*PKN2*^flox/flox^”) and Cre adenovirus (“Cre;*PKN2*^flox/flox^”), respectively. (A) Flow cytometry to detect cell surface expression of integrins. Cells were collected using trypsin-EDTA treatment and then cultured in a 2% agar coated flat bottom well plate for 3 h before starting flow cytometric analysis. Cells were resuspended in staining buffer (0.5% bovine serum albumin in Hanks’ Balanced Salt Solution with 25 mM HEPES, pH 7.5 and 0.1% sodium azide) and labeled with indicated primary antibodies for 30 min on ice. The monoclonal antibodies used for flow cytometric analysis of cell-surface antigens were allophycocyanin (APC)-conjugated anti-mouse CD49e (clone; 5H10-27; BioLegend) for detection of integrin α5 (“integrin α5”) and APC-conjugated anti-mouse/rat CD29 (clone; HMβ1-1; BioLegend) for detection of integrin β1 (“integrin β1”). After washing with staining buffer, cells were incubated with 7-amino-actinomycin D for 15 min at room temperature. Cells were analysed by flow cytometry using an Accuri™ system (BD Biosciences, Franklin Lakes, NJ, USA).  Cell surface expression of integrin α5 and integrin β1 was evaluated by mean fluorescent intensity. The mean fluorescent intensity was calculated using BD Accuri™ C6 software (BD Biosciences, Franklin Lakes, NJ, USA). Data were analysed with paired t-tests. n = 4. NS, not significant. (B) Immunoblotting of integrins. After removing culture medium, whole cell lysate was subjected to immunoblotting.

**Supplementary Fig. 4. PKN2 depletion does not affect mRNA expression of *Slug*, *Twist1* and *ZEB1*, whereas *c-Myc* expression is increased in adherent cells.**

*PKN2*^flox/flox^ cells were incubated for 48 h after treatment with mock adenovirus (“*PKN2*^flox/flox^”) and Cre adenovirus (“Cre;*PKN2*^flox/flox^”), respectively. mRNA expression of *Slug*, *ZEB1*, *Twist1* and *c-Myc* was measured by RT-qPCR. Relative gene expression was calculated by delta-delta CT method. Expression of *GAPDH* mRNA was used as an internal control for normalization. Data were analysed by unpaired t-test. n = 3. *P < 0.05, NS, not significant.

[1] H. Mukai, M. Miyahara, H. Sunakawa, et al., Translocation of PKN from the cytosol to the nucleus induced by stresses, Proc Natl Acad Sci U S A 93.19. (1996) 10195-10199.

[2] H. Mukai, A. Muramatsu, R. Mashud, et al., PKN3 is the major regulator of angiogenesis and tumor metastasis in mice, Sci Rep 6 (2016) 18979.
