# Supplementary figures and images for: PKN2 is involved in aggregation and spheroid formation of fibroblasts in suspension culture by regulating cell motility and N-cadherin expression
Source: Biochem Biophys Rep. 2021 Jan 2;25:100895. doi: 10.1016/j.bbrep.2020.100895 (PMC7787963; doi:10.1016/j.bbrep.2020.100895)

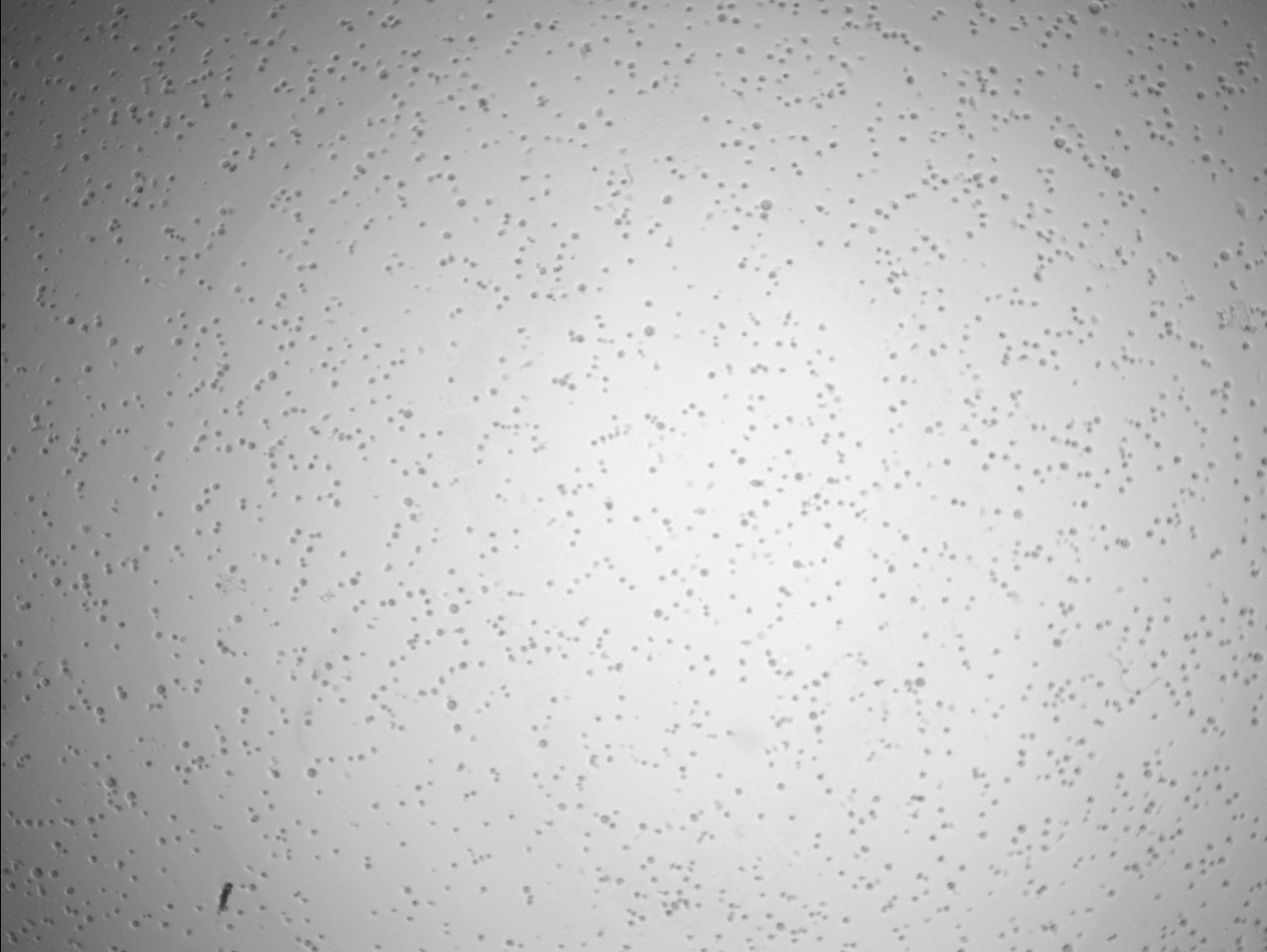

Supplement: Multimedia component 1 [file mmc1.zip › Still Movie 2.tif]

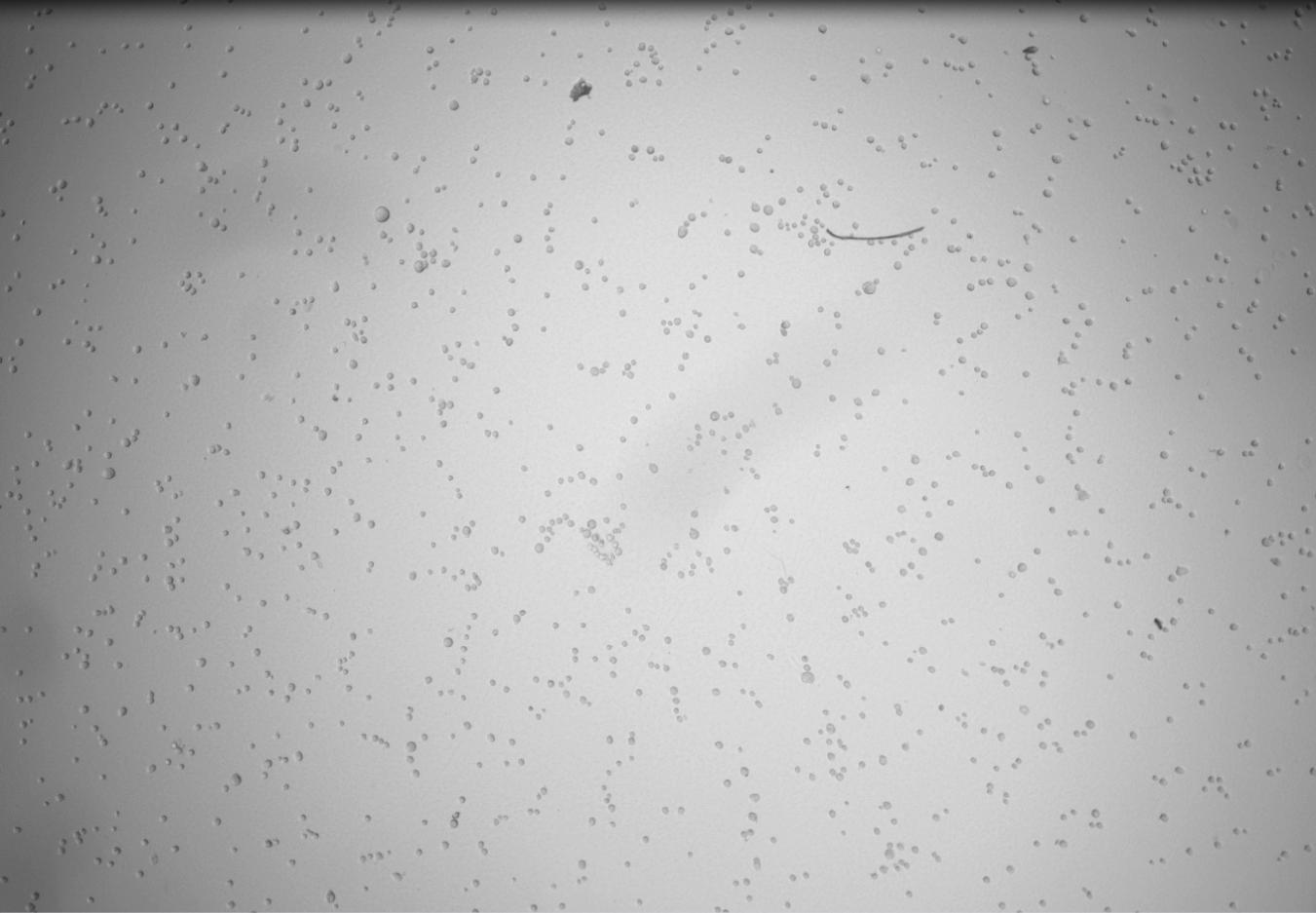

Supplement: Multimedia component 1 [file mmc1.zip › Still Movie 1.tif]
